# KRAS Early Testing: Consensus Initiative and Cost-Effectiveness Evaluation for Metastatic Colorectal Patients in an Italian Setting

**DOI:** 10.1371/journal.pone.0085897

**Published:** 2014-01-20

**Authors:** Carlo Barone, Carmine Pinto, Nicola Normanno, Lorenzo Capussotti, Francesco Cognetti, Alfredo Falcone, Lorenzo Mantovani

**Affiliations:** 1 Medical Oncology Unit, Gemelli Hospital, Rome, Italy; 2 Medical Oncology Unit, S.Orsola-Malpighi Hospital, Bologna, Italy; 3 Cellular Biology and Biotherapies Unit, Pascale Foundation, Naples, Italy; 4 General and Oncological Surgery, Umberto I Hospital, Turin, Italy; 5 Medical Oncology Department, Regina Elena Hospital, Rome, Italy; 6 Oncology, Transplantations and New Medical Technologies Department, Santa Chiara Hospital, Pisa, Italy; 7 Clinical Medicine and Surgery Unit, Federico II University of Naples, Naples, Italy; Centro di Riferimento Oncologico, IRCCS National Cancer Institute, Italy

## Abstract

KRAS testing is relevant for the choice of the most appropriate first-line therapy of metastatic colorectal cancer (CRC). Strategies for preventing unequal access to the test should be implemented, but their relevance in the practice is related to economic sustainability. The study adopted the Delphi technique to reach a consensus on several topics. Issues related to execution of KRAS testing were identified by an expert’s board and proposed to 108 Italian oncologists and pathologists through two subsequent questionnaires. The emerging proposal was evaluated by decision analyses models employed by technology assessment agencies in order to assess cost-effectiveness. Alternative therapeutic strategies included most commonly used chemotherapy regimens alone or in combination with cetuximab or bevacizumab. The survey indicated that time interval for obtaining KRAS test should not exceed 15 days, 10 days being an optimal interval. To assure the access to proper treatment, a useful strategy should be to anticipate the test after radical resection in patients at high risk of relapse. Early KRAS testing in high risk CRC patients generates incremental cost-effectiveness ratios between 6,000 and 13,000 Euro per quality adjusted life year (QALY) gained. In extensive sensitivity analyses ICER’s were always below 15,000 Euro per QALY gained, far within the threshold of 60,000 Euro/QALY gained accepted by regulatory institutions in Italy. In metastatic CRC a time interval higher than 15 days for result of KRAS testing limits access to therapeutic choices. Anticipating KRAS testing before the onset of metastatic disease in patients at high risk does not affect the sustainability and cost-effectiveness profile of cetuximab in first-line mCRC. Early KRAS testing may prevent this inequality in high-risk patients, whether they develop metastases, and is a cost-effective strategy. Based on these results, present joined recommendations of Italian societies of Oncology and Pathology should be updated including early KRAS testing.

## Introduction

Since 2008 KRAS mutational status has become the main tissue biomarker of resistance to anti-EGFR monoclonal antibodies in metastatic colorectal cancer (CRC). Large phase III and randomized phase II studies have demonstrated that mutation of KRAS gene predicts resistance to treatment with an anti-EGFR antibody either alone or in combination with chemotherapy [1], [2]. The effect of combination of anti-EGFR antibody and chemotherapy seems particularly relevant in patients with liver limited initially not resectable disease [3], [4]. Furthermore, in palliative setting the addition of anti-EGFR monoclonal antibodies to chemotherapy regimens increases the progression free survival and overall survival [5]. Only in few studies biased by selection and methodological issues the predictive role of KRAS has not clearly emerged; however, also in one of these studies response rate was significantly higher in wild-type (wt) tumors [6], [7].

The most accepted guidelines now consider KRAS mutation status a central step of decision-making process in the therapy of metastatic CRC, both in potentially resectable and palliative treatment settings. Access to the advantage provided by this test, however, may be limited by some practical difficulties that may hinder the therapeutic opportunities resulting from anti-EGFR targeting therapy. Mutational analysis, in fact, needs technical and expertise resources that may be not everywhere available [8]. Both complexity of the test and different access to molecular biology laboratory may cause delays, which often conflict with the urgency of clinical decision. This means that an unknown, but not irrelevant, percentage of patients is excluded from a potential therapeutic advantage with negative consequences either on symptom control or resection rate and survival.

**Figure 1:**
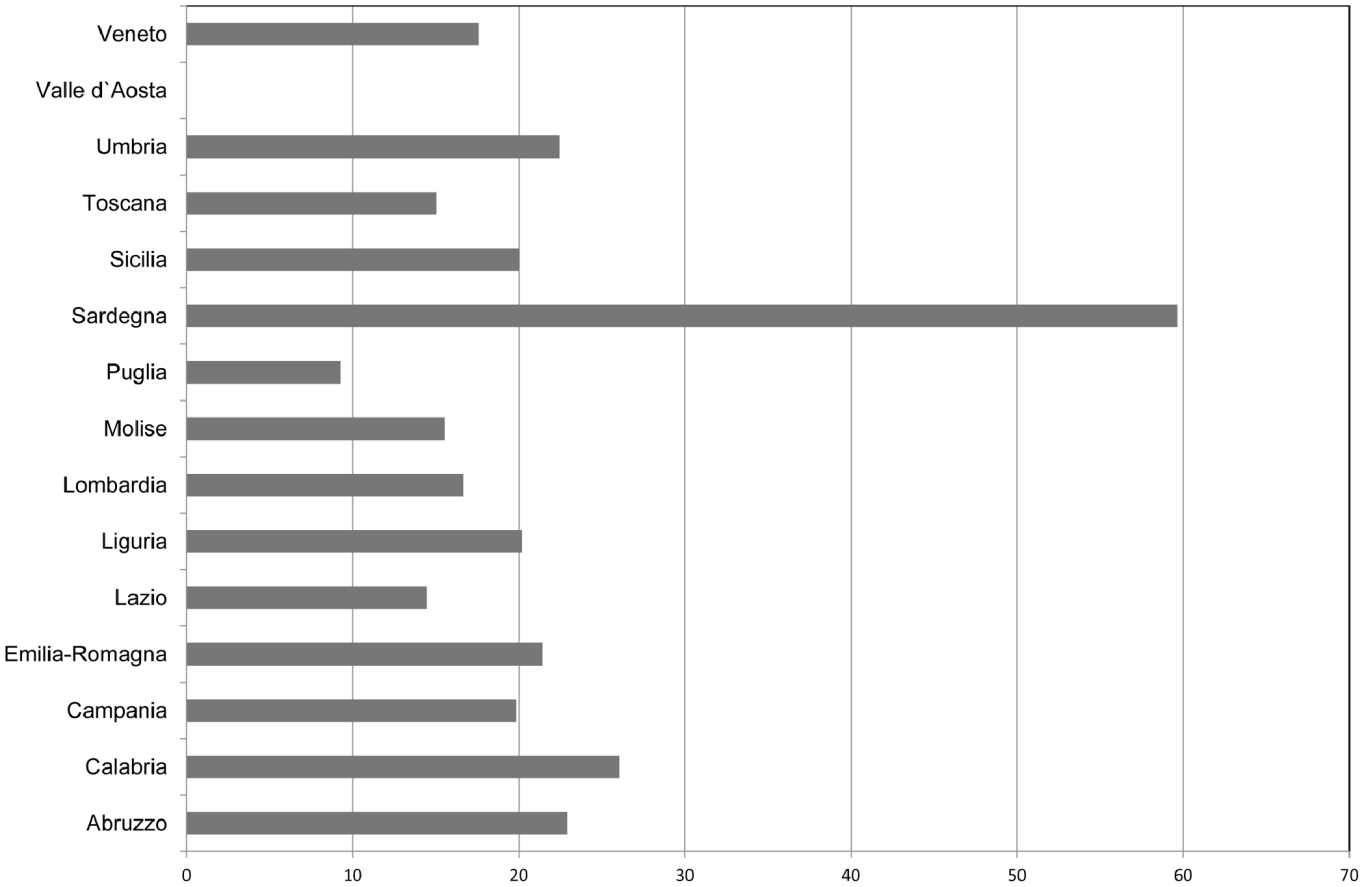
KRAS aKtive program in Italy – Requested time (days) to diagnosis –2011.

The peculiarity of the Italian situation can be clearly seen from the data collected with the KRAS aKtive system, a network aimed to facilitate KRAS testing in Italy. In the period from 1 January 2011 to 31 December 2011, this project has involved 407 oncologists and 125 pathologists, and 24 reference laboratories in 16 of the 21 Italian regions [9]. The times to the diagnosis in most of involved regions result more than 15 days: with the exception of the only region of Puglia, all other times are longer than 10 days and, except Lazio and Tuscany, all are over 15 days (Figure 1). This value is similar to that found in a retrospective study including 160 French centers, where the median time between prescription and result of the K-RAS testing was 19 months [10].

In addition to differences in reaching the needed resources for KRAS test, economic concerns may have the potential to constrain the use of anti-EGFR antibodies, despite the large evidence resulting from clinical trials. Recently, a health technology assessment report has evaluated the clinical and economic profile of cetuximab in first-line metastatic colorectal cancer (mCRC) in Italy on a specific population (KRAS wt liver limited disease patients). The economic analysis of cetuximab in the treatment of metastatic colorectal cancer shows that this therapy, in combination with FOLFOX and FOLFIRI, is more cost-effective than the alternatives currently available in first line (bevacizumab+FOLFOX, FOLFOX, FOLFIRI); therefore cetuximab can be considered as a sustainable alternative for the NHS [11].

Among the possible strategies, aimed to equalize patients opportunity of access to the potential advantage of anti-EGFR strategy, the anticipation of KRAS testing might be of value. In the present study we have analyzed the opinion and the attitude of a representative group of Italian specialists, mainly oncologists and pathologists, on the implication of KRAS testing in order to optimize overall therapeutic approach to patients with metastatic CRC. The economic supportability of a different strategy of KRAS testing was assessed by a specifically modeled cost-effectiveness analysis.

## Methods

### Delphi Technique

The Delphi technique is a validated consensus-building process to develop consensus and make group-based decisions in a variety of fields [12], [13]. It was conceived and developed in the mid-1950s by researchers at the Rand Corporation as a way to predict the impact of technologies or interventions on complex systems, which has often been used in the social and health care context [14]–[16].

**Figure 2:**
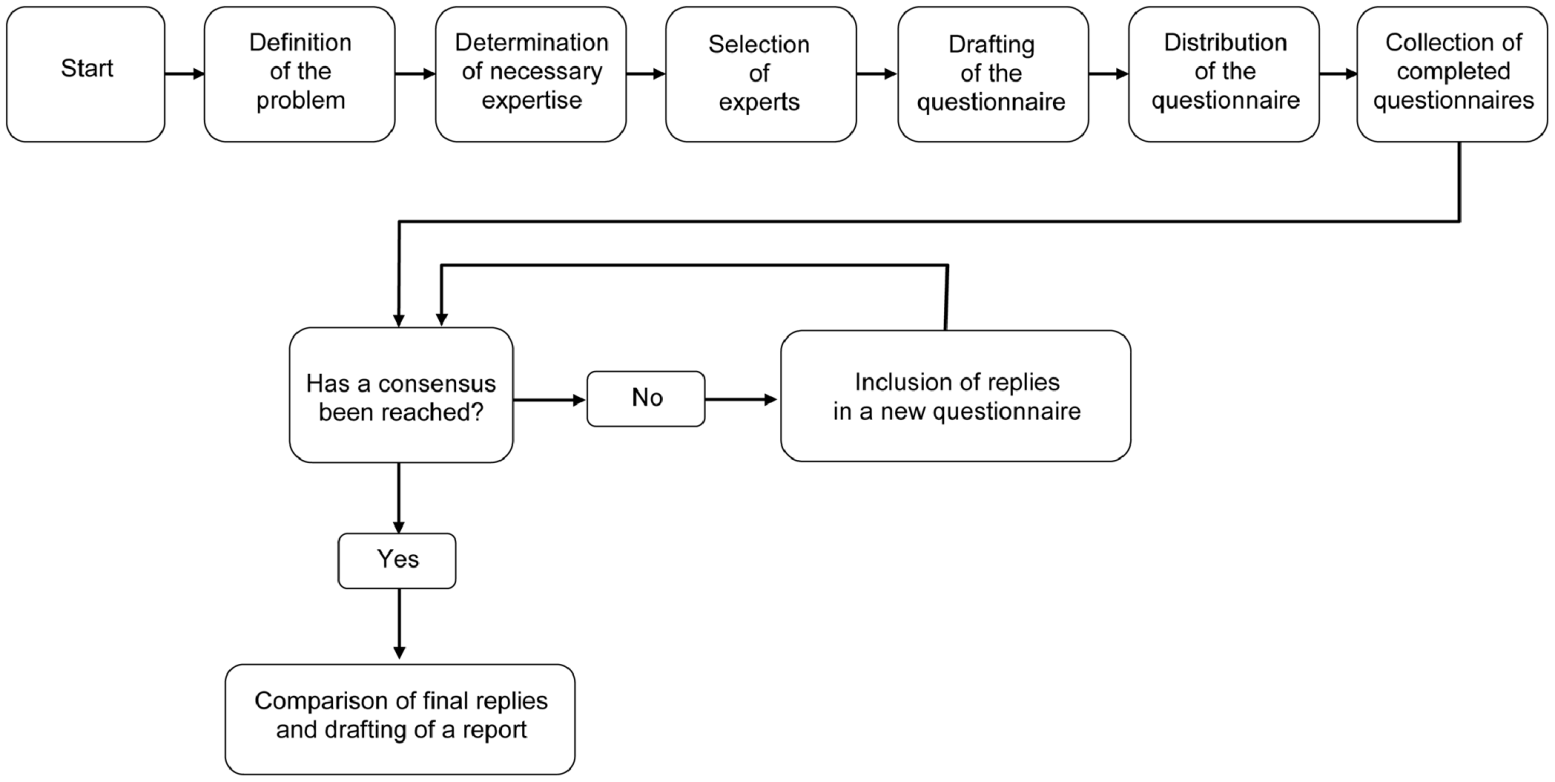
Phases of the Delphi method.

The Delphi method (Figure 2) is traditionally based on three fundamental concepts. The first is anonymity. The participants never meet each other during the process. Each participant submits his or her opinion independently, by completing a specially designed questionnaire. The replies are then disclosed to all participants, without identifying the particular respondent. The second concept is controlled feedback. The process consists of several rounds; during each of them the respondents are asked to judge all the opinions expressed in the previous rounds, often presented in the form of statistics. The last concept is statistical group response. The Delphi method reaches a collective decision and expresses it in terms of a statistical score. In addition to these basic characteristics, the Delphi method can be described as follows:

- It requires individual effort for the expression of an opinion.
- It requires written answers to questionnaires.
- The individual opinions (questionnaire responses) are collected and assembled by the project coordinator.
- The respondents have enough time to come up with and evaluate opinions (unlike task force meeting, in which, quite often, not enough time is allowed to assess other people’s opinions).

Delphi method was used specifically in the field of oncology and diagnostic test assessment for epidemiological, inhabitant awareness and behavior, and to set up and define healthcare protocols and procedures and treatment guidelines [17]–[27].

For the purpose of this specific project, a small group of specialists, the board of experts (comprised of six major specialists selected from Italian universities and public hospitals and one pharmacoeconomist) examined the scientific literature and developed a 22-item questionnaire (Q1; see Table 1), including 4 demographic initial questions. The questionnaire was designed for an expert panel consisting of 108 oncologists, pathologists, molecular biologists, surgeons, quite all of them persons in charge of hospital departments, randomly selected from different Italian regions.

**Table 1:** Questionnaire 1.

QUESTION	% alloptions	% combinedoptions (0–4/5–9)	Consensus
**5. In the institution where you work, the laboratory conducting the KRAS test:**			
a. is the same pathological anatomy and is based in your own hospital	47.66%		
b. is different from the pathological anatomy and is based in your own hospital	15.89%		
c. is the same pathological anatomy based in a different seat from your hospital but in thesame local health structure	2.80%		
d. is the same pathological anatomy based in a different seat from your hospital and in adifferent local health structure	7.48%		
e. is different from the pathological anatomy based in a different seat from your hospital but in thesame local health structure	1.87%		
f. is different from the pathological anatomy based in a different seat from your hospital and in adifferent local health structure	22.43%		
g. other (specify)	1.87%		
**6. In the institution where you work, the time elapsing from the request of KRAS testing** **to obtainment of the results is:**			
a. ≤7 days	26.92%		
b. 8–14 days	43.27%		
c. 15–21 days	23.08%		
d. 22–28 days	5.77%		
e. >28 days	0.96%		
**7. Do you think that the time required to obtain the response in a patient with metastatic** **CRC who is to receive a first line chemotherapy:**			
a. does not affect treatment choices	39.58%		
b. negatively affects treatment choices	19.79%		
c. limits the first line use of anti-EGFR monoclonal antibodies	38.54%		
d. other (specify)	2.08%		
**8. The best time to take the test for the detection of KRAS is at the diagnosis of** **potentially resectable liver metastases.**			**NO**
0 total disagreement	9.18%		
1	4.08%		
2	8.16%	33.67%	
3	10.20%		
4	2.04%		
5	6.12%		
6	8.16%		
7	9.18%	66.33%	
8	14.29%		
9 complete agreement	28.57%		
**9. The best time to take the test for KRAS is after radical resection of colorectal cancer in patients at high risk of relapse in order to have the data at the onset of metastases.**			**YES**
0 total disagreement	4.04%		
1	3.03%		
2	7.07%	26.26%	
3	9.09%		
4	3.03%		
5	6.06%		
6	7.07%		
7	7.07%	73.74%	
8	15.15%		
9 complete agreement	38.38%		
**10. If you believe that the time for the determination of KRAS mutations is inadequate,** **which are the causes that produce this delay you deem most important? (ranking 1 to 6)**	**Score +**	**Rank**	
a. Technical problems of the laboratory	211	1	
b. Secretarial/bureaucratic issues	209	2	
c. Availability of the material in the archive	180	4	
d. Finding material in the archive	192	3	
e. Sample preparation for testing	164	5	
f. Sending the sample to be examined	192	3	
g. Other (specify)	12	6	
**11. If the waiting time of KRAS test results are too long, the oncologist may decide the therapeutic strategy even without considering the possibility of a personalized therapy.**			**NO**
0 total disagreement	12.63%		
1	7.37%		
2	14.74%	55.79%	
3	9.47%		
4	11.58%		
5	10.53%		
6	9.47%		
7	11.58%	44.21%	
8	5.26%		
9 complete agreement	7.37%		
**12. In patients with CRC and potentially resectable liver metastases susceptible to a conversion therapy, waiting times for KRAS test results must be adequate to ensure the oncologist the possible use of an anti-EGFR monoclonal antibody in combination with chemotherapy.**			**YES**
0 total disagreement	1.04%		
1	0.00%		
2	0.00%	2.08%	
3	1.04%		
4	0.00%		
5	4.17%		
6	1.04%		
7	7.29%	97.92%	
8	16.67%		
9 complete agreement	68.75%		
**13. The evaluation of KRAS allows an efficient use of resources, ensuring in this way the selection the most appropriate and targeted treatment.**			**YES**
0 total disagreement	0.00%		
1	0.00%		
2	0.00%	1.01%	
3	0.00%		
4	1.01%		
5	0.00%		
6	1.01%		
7	14.14%	98.99%	
8	24.24%		
9 complete agreement	59.60%		
**14. The evaluation of KRAS in the primary tumour in patients at high risk is a financially sustainable strategy allowing the prompt use of the most appropriate therapy when the patient metastasizes.**			**YES**
0 total disagreement	3.03%		
1	4.04%		
2	7.07%	25.25%	
3	7.07%		
4	4.04%		
5	4.04%		
6	3.03%		
7	14.14%	74.75%	
8	20.20%		
9 complete agreement	33.33%		
**15. There is sufficient scientific literature that defines the operated patient at high risk of relapse.**			**YES**
0 total disagreement	0.00%		
1	0.00%		
2	1.05%	14.74%	
3	6.32%		
4	7.37%		
5	9.47%		
6	15.79%		
7	24.21%	85.26%	
8	22.11%		
9 complete agreement	13.68%		
**16. In radically operated patients N0 at diagnosis, what are the prognostic factors to be taken into account?** (ranking 1 to 6)	Score +	Rank	
a. pT4	394	1	
b. Grading	306	2	
c. Lymph angioinvasion	271	4	
d. Mucinous histotype	184	5	
e. Intestinal occlusion-perforation	297	3	
f. Value of CEA	126	6	
g. Other (specify)	21	7	
**17. In radically operated patients N+ at diagnosis, what are the prognostic factors to be taken into account?** (ranking 1 to 7)	Score +	Rank	
a. pT4	376	2	
b. pN1	358	3	
c. pN2	502	1	
d. Mucinous histotype	213	6	
e. Lymph angioinvasion	280	4	
f. Value of CEA	137	7	
g. Intestinal occlusion-perforation	265	5	
h. Other (specify)	9	8	
**18. In patients with surgically removed isolated peritoneal carcinomatosis and/or positive peritoneal washing and/or removed ovarian metastases the determination for KRAS should always be performed.**			**YES**
0 total disagreement	4.30%		
1	0.00%		
2	3.23%	13.98%	
3	3.23%		
4	3.23%		
5	2.15%		
6	4.30%		
7	13.98%	86.02%	
8	21.51%		
9 complete agreement	44.09%		
**19. The determination of KRAS status is a key element in deciding the therapeutic strategy of patients with metastatic colorectal cancer.**			**YES**
0 total disagreement	0.00%		
1	0.00%		
2	0.98%	3.92%	
3	0.98%		
4	1.96%		
5	2.94%		
6	2.94%		
7	11.76%	96.08%	
8	21.57%		
9 complete agreement	56.86%		
**20. For the characterization of a colorectal tumour the evaluation of KRAS mutation status is the only molecular parameter to be considered in clinical practice.**			**NO**
0 total disagreement	8.82%		
1	4.90%		
2	4.90%	38.24%	
3	8.82%		
4	10.78%		
5	10.78%		
6	14.71%		
7	13.73%	61.76%	
8	10.78%		
9 complete agreement	11.76%		
**21. In clinical practice which molecular parameters do you use routinely for selecting the first line treatment in patients with metastatic colorectal cancer?**			
a. KRAS mutations (only exon 12, 13)	68	51.13%	
b. KRAS mutations (all mutations)	32	24.06%	
c. BRAF mutations	26	19.55%	
d. PI3K mutations	2	1.50%	
e. State of PTEN	3	2.26%	
g. Other (specify)	2	1.50%	
**22. The material for KRAS testing is either the primary tumour or the metastases.**			**YES**
0 total disagreement	1.96%		
1	0.00%		
2	3.92%	17.65%	
3	3.92%		
4	7.84%		
5	7.84%		
6	6.86%		
7	16.67%	82.35%	
8	13.73%		
9 complete agreement	37.25%		

Answers are expressed as percentage of all responses. Cut-off level to reach consensus: two-thirds (67%) of agreement of effective answers.

Q1 was written in order to avoid possible bias caused by inadvertently influencing replies, so the sequence of questions was ordered within a framework of three mains chapters (Management of patients with metastatic colorectal cancer; Definition of operated patient at high risk of relapse; Anticipation of KRAS testing at the diagnosis of primary tumor). For each question, space was provided for comments.

The majority of Q1 questions (11 out of 18) allowed a classified answer on a scale of 0 (total disagreement) to 9 (complete agreement) while other asked the responders either to rank a set of provided options in order of relevance following their clinical opinion, or to fill in open-ended questions.

To better analyze the replies, two categories of answers were defined for the questions with a 0–9 range:

- Score 0–4: negative answer;
- Score 5–9: positive answer.

After the replies to Q1 were processed, a second questionnaire (Q2) was developed (Table 2). Q2 was presented to the same expert panel and replies were collected and processed in the same way, as done with Q1.

**Table 2:** Questionnaire 2.

QUESTION	% all options	Consensus
**3. In the institution where you work, the time elapsing from** **the request of KRAS testing to obtainment of the results is:**		
a. ≤7 days	29.79%	
b. 8–14 days	44.68%	
c. 15–21 days	17.02%	
d. 22–28 days	6.38%	
e. >28 days	2.13%	
**4. Do you think that in a patient with metastatic CRC who is to receive a first line chemotherapy a** **time interval longer than 15 days for obtaining the response could limit the therapeutic choices?**		**YES**
a. Yes	74.73%	
b. No	25.27%	
**5. If so, what do you think is the maximum waiting time for KRAS test results to proceed with** **a therapy?** (in days)		
Average	10	
Median	9	
**6. Do you think that the best time to take the test for KRAS is at the onset of the metastatic disease?**		**NO**
a. Yes	60.22%	
b. No	39.78%	
**6.a. If no, which you think is the best time?**		
At diagnosis, especially in patients at high risk of relapse (10)	40%	
At the surgery, in patients at high risk of recurrence (13)	52%	
**6.b. Do you think it is all the way useful to anticipate it?**		**YES**
a. Yes	72.04%	
b. No	27.96%	
**7. If the waiting time for KRAS test results are too long, should the oncologist decide to go ahead** **with alternative therapeutic strategies giving up the opportunity to use a personalized therapy?**		
a. Yes	51.19%	
b. No	48.81%	
**7.a. Do you think that the possible treatment choice alternative to personalized therapy is** **equally effective?**		
a. Yes	36.14%	
b. No	63.86%	
**8. Is the evaluation of KRAS in the primary tumour in patients at high risk, so to promptly use the most** **appropriate therapy when the patient metastasizes, a sustainable strategy?**		**YES**
a. Yes	74.47%	
b. No	25.53%	
**8.a. If no, please state if this is due to:**		
a. Organizational reasons	32.35%	
b. Financial reasons	67.65%	
**9. In the light of the anti-EGFR filed indications and of the supporting scientific data, do you think** **that the KRAS is the only molecular parameter to consider in clinical practice?**		**NO**
a. Yes	43.62%	
b. No	56.38%	

Answers are expressed as percentage of all responses. Cut-off level to reach consensus: two-thirds (67%) of agreement of effective answers.

After both rounds, the level of agreement was evaluated based on the percentage of positive answers to each question. To reach consensus, a cut-off level of two-thirds (67%) of agreement (for positive or negative answers) was required for the first (Q1) and the second round (Q2). These arbitrary but standard consensus levels were agreed on by all members of the board before beginning the study.

### Cost-effectiveness Analysis

In order to assess the cost-effectiveness of early KRAS testing in high-risk non metastatic CRC patients, we adapted a model previously used to assess the cost effectiveness of cetuximab plus chemotherapy *vs* chemotherapy alone or chemotherapy plus bevacizumab, i.e. the only other biologic agent to date approved in Italy in first line colorectal cancer [11]. The model has been developed and adapted based on models previously used in assessments performed by technology assessment agencies, e.g. NICE [28] and SMC [29].

Alternative therapeutic strategies included chemotherapy regimens (FOLFOX4, FOLFIRI), and biological drugs in combination with chemotherapy (cetuximab+FOLFOX-4, cetuximab+FOLFIRI, bevacizumab+FOLFOX4). The models were adapted to take into account for early KRAS testing in high-risk patients in those cases in which a deferred test would impair a well-timed treatment including cetuximab. The models have been populated with Italy specific cost data (drugs, tests, hospital admissions, administration, toxicity management, etc.), incorporating patients’ access schemes (cost/risk sharing agreements and capping) as described in details in the SMC assessment [29].

Not all patients at high risk would eventually develop metastases and would therefore be candidate for treatment with cetuximab. The cost effectiveness of early KRAS testing mimics the situation in which part of KRAS test would be performed in vain (i.e. for those patients who would not eventually develop metastases), and part would only be anticipated (i.e. for those who would subsequently develop metastases). Computationally, this is equivalent to adding to the population of patients eventually developing metastatic disease and being treated, the cost of KRAS tests of the population of patients that would not develop metastases and for which the test would have not been necessary. The size of these two populations depends on the level of risk of developing metastatic disease. In order to simulate the incremental cost effectiveness that would be generated by early KRAS testing in high risk patients and to test the robustness of results, we conducted several analyses under different assumptions, by setting the level of risk of developing metastatic disease at the level of 1 patients out of 2 (i.e. 50% chance), 1/3, 1/4, 1/5 and 1/10, i.e. including very low risk. The cost of KRAS test is quantified at 160 Euro per test. Costs are expressed in Euro of 2012 and effects are expressed in quality adjusted life years (QALY) gained.

## Results

Q1 was sent to 160 specialists involved in diagnosis and treatment process in the oncology field: 17 molecular biologists (10.7%), 21 surgeons (13.1%), 85 oncologists (53.1%), 37 pathologists (23.1%). These 160 specialists define a representative sample of the different Italian regions. The 108 respondents (67.5% of those initially selected) were from northern (43%), central (28%) and southern (29%) Italy. Q2 was sent to the 108 responders to Q1, and 96 (88.8% of them) returned an answer.

The percentage of respondents choosing each reply is presented in tables 1 and 2. By analyzing and evaluating the replies to both questionnaires, the board identified the following statements about different issues on KRAS testing that attained expert agreement of 67% or more:

### Management of Patients with Metastatic Colorectal Cancer

1. The determination of KRAS status is a key element in deciding the therapeutic strategy of patients with metastatic CRC (Q1–19)
2. In clinical practice, the molecular parameters used routinely for the selection of first choice treatment in patients with metastatic CRC are primarily KRAS mutations in exons 12 and 13 (Q1–21)
3. KRAS evaluation allows for an efficient use of resources, favoring the choice of the most appropriate and targeted treatment (Q1–13; Q2–8)
4. Both primary tumor and metastases may be appropriate for KRAS testing (Q1–22)
5. In a patient with metastatic CRC who must undergo a first-line chemotherapy, a time interval of more than 15 days for the result of KRAS testing limits the therapeutic choices (Q1–7; Q2–4)
6. The maximum acceptable time for KRAS test result should not exceed 10 days (Q2–5)
7. In patients with CRC and potentially resectable liver metastases susceptible of conversion therapy, waiting time for KRAS test result must be adequate in order to ensure the eventual use of a monoclonal anti-EGFR antibody in combination with chemotherapy (Q1–12)
8. If waiting time for KRAS test result is too long, a therapeutic strategy alternative to the personalized therapy is not equally effective (Q1–11; Q2–7; Q2–7a)

### Definition of Colorectal Cancer Operated Patients at High Risk of Relapse

1. In radically resected N0 patients, the most significant prognostic factors are pT4, tumor grade, intestinal occlusion/perforation at presentation (Q1–16)
2. In radically resected N+ patients, the most significant prognostic factors are pN2 and pT4 (Q1–17)
3. Patients with surgically removed limited peritoneal carcinomatosis and/or positive peritoneal washing and/or removed ovarian metastases always require KRAS determination (Q1–18)

### Anticipation of KRAS Testing at the Diagnosis of Primary Tumor

1. In patients at high risk of relapse anticipating KRAS testing before the onset of metastatic disease may allow the timely use of the most appropriate therapy when the patients metastasizes (Q1–14; Q2–6b; Q2–8)
2. In patients at high risk of relapse, the best time for KRAS testing is after radical resection of CRC in order to have the information when it should be necessary (Q1–9)
3. Anticipating KRAS testing before the onset of metastatic disease in patients at high risk does not affect the sustainability of cetuximab in first-line mCRC regardless of the level of risk of developing metastases (Q1–14; Q2–8)

Cost effectiveness results of early KRAS testing in high risk patients that would have no access to well-timed KRAS testing if they develop metastatic disease are summarized in Table 3.

**Table 3:** Cost effectiveness results of Early KRAS testing in high risk patients that would have no access to well-timed KRAS testing if they develop metastatic disease.

Treatment of choice	Expected cost	Expected QALY*	ICER*	ICER 1	ICER 2	ICER 3	ICER 4	ICER 5
Cetuximab+FOLFOX-4	29.557	2,92						
FOLFOX-4	17.643	2,01	13.092,31	13.268,13	13,443,96	13.619,78	13.795,60	14,674,73
Bevacizumab+FOLFOX-4	25.128	2,12	5.536,25	5.736,25	5.936,25	6.136,25	6.336,25	7.336,25
Cetuximab+FOLFIRI	29.154	2,94						
FOLFIRI	15.983	1,60	9.829,10	9.948,51	10.067,91	10.187,31	10.306,72	10.903,73
ICER: Incremental Cost Effectiveness Ratio, calculated as incremental cost per QALY gained								
*Adapted from Barone *et al*, 2012 [11].								
ICER 1 = Incremental Cost Effectiveness Ratio: risk 1/2								
ICER 2 = Incremental Cost Effectiveness Ratio: risk 1/3								
ICER 3 = Incremental Cost Effectiveness Ratio: risk 1/4								
ICER 4 = Incremental Cost Effectiveness Ratio: risk 1/5								
ICER 5 = Incremental Cost Effectiveness Ratio: risk 1/10								

ICER of cetuximab+FOLFOX *vs* bevacizumab+FOLFOX is lower than that of cetuximab+FOLFOX *vs* FOLFOX due to the limited advantage of bevacizumab+FOLFOX on FOLFOX. Differential cost of cetuximab+FOLFOX *vs* bevacizumab+FOLFOX is minimal whereas efficacy favours cetuximab+FOLFOX on FOLFOX.

In this table the treatment with cetuximab+FOLFOX-4 is compared to FOLFOX-4 and bevacizumab+FOLFOX-4, while cetuximab+FOLFIRI is compared to FOLFIRI. Anticipating the KRAS testing has as consequence an increase of the costs for the metastatic population represented by the cost of KRAS tests of those patients that will not develop metastases and for which the test would not be necessary. In order to simulate the incremental cost effectiveness that would be generated by early KRAS testing in high risk patients and to test the robustness of results, we conducted several analyses under different assumptions, by setting the level of risk of developing metastatic disease at the level of 1 patients out of 2 (i.e. 50% chance), 1/3, 1/4, 1/5 and 1/10, i.e. including very low risk. The range of ICER generated is between 9,948.51 Euro/QALY (ICER1) and 10,903.73 Euro/QALY (ICER5), far within the threshold of 60,000 Euro/QALY gained accepted by regulatory institutions in Italy.

## Discussion

The Delphi method used in the present analysis has advantages and drawbacks. The methodology can overcome many of the limitations intrinsic to traditional group decision-making processes, it keeps attention directly on the issue, and it is flexible and inexpensive compared to focus group. Depending on the nature of the problem, the method can be adjusted for improved overall efficacy. Since the use of strict statistical methods for setting guidelines is rather problematic, due to the quantitative nature of the expected results, the use of modified classification procedures makes easier monitoring and expression of the process by which a consensus is developed. On the other hand, there are some disadvantages: information comes from a selected group of people and may be not representative; it tends to eliminate extreme positions and to force a middle-of-the-road consensus; it is more time-consuming than group process methods; it requires skills in written communication; finally, it requires adequate time and participant commitment.

At the end, the consensus process has become part of the technology for solving problems in health service and medicine by putting the knowledge and experience of practitioners and other experts in touch with scientific literature [30].

In advanced CRC the treatment strategy based on KRAS status is related to clinical outcome of patients [3], [5]. Determining the tumor K-RAS status is now part of clinical practice in most countries; for example, in France the prescription rate in first line therapy is estimated around 81% [10]. In the present study most of experts come from centers certified by the national quality program for KRAS testing or participating to the KRAS aKtive program, so their opinion reflects both the clinical attitude and the real situation in treatment of CRC cancer in Italy. They strongly agree on the relevance of KRAS mutational status in the decision process in metastatic CRC because it allows both the choice of the most appropriate treatment and the proper use of resources. Although other mutations in KRAS, BRAF and PIK3CA genes have been evaluated in relation to resistance to anti-EGFR antibodies, only the most frequent seven KRAS mutations of exons 12 and 13 (Gly12Ala, Gly12Asp, Gly12Arg, Gly12Cys,Gly12Ser, Gly12Val, Gly13Asp) have been investigated in phase III clinical trials and have a proven relation to clinical efficacy of anti-EGFR antibodies [5], [31]. Accordingly, most panelists in our analysis believe that only these mutations should be considered in the practice and that accuracy in the determination of the test is not affected by the source of the sample, either the primary tumor or metastases.

Despite about 40% of experts think that time required to obtain the result of KRAS test do not affect treatment choice in first-line treatment of metastatic CRC, based on their experience, most of them doubt that presently an alternative therapeutic choice might have the same level of effectiveness when the possibility of personalizing treatment were deemed. In addition, large percentage of panelists agree that in first-line chemotherapy a time interval longer than 15 days for obtaining the result actually could limit the therapeutic choices. In support of this view, an interval of 10 days has been considered an optimal time, suggesting that waiting longer is not rare. As a matter of fact, in the previously mentioned KRAS aKtive system it has been shown that delay in obtaining KRAS testing is more than 15 days in most of Italian regions [9].

The clinical situation, however, seems to influence the relevance of shortening the time needed for having test results. In fact, a very large percentage of panelists agree that, in conversion therapy of liver metastases, waiting time for KRAS test must allow the use of a monoclonal anti-EGFR antibody in combination with chemotherapy. This partial divergence may be the consequence of two different aspects: on the one hand, response and tumor shrinkage is largely perceived as important in conversion therapy, as it results in several clinical studies. On the other hand, Italian regulatory rules allow to use cetuximab later whether it was not used in first-line. Therefore, some oncologists might conceive that, when response does not seem to have a crucial role, the activity of cetuximab might be recovered in a subsequent line of treatment. However, this thinking does not consider that a number of KRAS positive patients not responding to an alternative treatment might be definitively excluded from EGFR targeting therapy due to progressive disease, deterioration of performance status and eventually death.

In the metastatic CRC, only when the mutational status of KRAS is known, a proper therapeutic decision may be reached. Panelists agree that in patients with metachronous metastases this may be undoubtedly realized by anticipating KRAS test before the onset of metastatic disease, meeting the need of improving decision making in clinical practice, as it has been recently underlined [10]. In the opinion of most experts the right time for KRAS test is after radical resection of CRC. This implies the need of identifying which group of patients should be considered at high risk of relapse. Panelists largely shared that conditions as surgically removed limited peritoneal carcinomatosis and/or positive peritoneal washing and/or removed ovarian metastases have a very high risk of relapse and require an immediate assessment of KRAS mutational status. In radically resected patients, according to existing evidence, other unfavorable prognostic factors have been recognized including: pT4, high tumor grade, intestinal occlusion-perforation at presentation in N- patients, and pN2 followed by pT4 in N+.

Contrary to what happens in other European countries, such as Spain where a network do exist but all tests are referred to only five laboratories [32], in Italy the system is more scattered in the national territory [9] and the perception of relevance of the timing of KRAS testing is diffuse. Therefore, the convergence of panelists opinion toward the anticipation of KRAS testing in order to ensure the timely use of the most appropriate and personalized therapy in metastatic disease means that an anti-EGFR therapy should be extensively considered in metastatic KRAS wt tumors. As a consequence, the economic impact and sustainability of such a strategy has been evaluated.

KRAS testing to limit use of EGFR inhibitors to patients with KRAS wild-type tumors resulted in net savings of $7,500 to $1,200 and of €3,900 to €9,600 per patient in the United States and Germany, respectively, and was shown cost-effective in patients with KRAS wild-type liver limited disease [33], [34]. It has been estimated that KRAS testing to all metastatic CRCs may realize annual savings ranging from $740 to $103 million in the United States depending on parameters and alternative treatment included in the analysis, that result in different outcomes in terms of cost-effectiveness [35], [36].

Our analysis was aimed to evaluate the different scenery of KRAS early testing not in metastatic CRCs but in patients with CRC at risk of relapse and/or metastases. Not all patients at high risk will eventually develop metastases and will therefore be eligible for treatment with cetuximab. The cost effectiveness of early KRAS testing mimics the situation in which part of KRAS test will be performed in vain (i.e. for those patients who will not eventually develop metastases), and part will only be anticipated (i.e. for those who will subsequently develop metastases). Computationally, this is equivalent to adding to the population of patients eventually developing metastatic disease and being treated, the cost of KRAS tests of the population of patients that will not develop metastases and for which the test will not be necessary. The results of this economic analysis on early KRAS testing in CRC patients, including those at high as well those at very low risk, show that the incremental cost-effectiveness ratio remains within the range of 6,000 to 15,000 Euro per quality adjusted life year (QALY) gained, regardless of the level of risk of developing metastases and far within the threshold of 60,000 Euro/QALY gained accepted by regulatory institutions in Italy (Table 3). In our analysis, we hypothesized that the cost of promptly treating patients developing metastases will include the cost of those KRAS tests performed in patients that would not develop metastases. In case of absence of timely knowledge on KRAS status, patients may receive alternative treatments, which may be highly variable from area to area, but it will essentially include a mix of FOLFOX/FOLFIRI with or without bevacizumab. Therefore, in order to allow the decision maker to judge based on the actual practice in any relevant area, we assessed early KRAS testing against any single alternative therapeutic strategy that may take place in absence of adequate knowledge on KRAS status. In this way, every decision maker can find information on each possible relevant comparator assessed one by one against early KRAS test. In other words, rather than assessing the “mix” of treatments that patients may receive as a whole, we assessed each single part of the mix, one by one against KRAS testing.

This study shows that anticipating KRAS testing in patients with high risk of relapse is a strategy accepted and perceived as useful by Italian specialists in the field of diagnosis and treatment of CRC. A specifically modeled cost-effectiveness analysis suggests that this clinical attitude is economically sustainable.

Taken together, the economic sustainability and the convergence of panelists toward the anticipation of KRAS testing support the inclusion of early KRAS testing in the joined recommendations of societies of Medical Oncology and Pathology in Italy. This might represent the necessary condition for a larger national program of guidelines in molecular diagnosis of cancer.

### Glossary

#### Cost-Effectiveness Analysis (CEA)

Analysis comparing the costs and clinical outcomes of at least two therapeutic alternatives (one of which may also be the non-treatment). Outcomes (benefits) resulting from the alternatives are expressed in clinical units (e.g. years of life gained, number of lives saved, reduction in the incidence of a disease).

#### Cost-Utility Analysis (CUA)

Analysis comparing the costs and the consequences of at least two alternatives (one of which can also be the non-treatment), capturing outcomes in terms of both quantity and quality of life (utility) simultaneously. The outcomes indicator generally used in a CUA is the QALY. The effects of medical treatment are expressed in terms of quality-adjusted life years gained (QALY).

#### Incremental Cost-Effectiveness Ratio (ICER)

Ratio between the difference in costs and the difference of the outcomes generated by the two alternatives (one of which may also be the non-treatment). It is measured in terms of economic investment required to achieve an incremental clinical benefit (Example: Euro/QALY).

#### Quality Adjusted Life Years (QALY)

Life year gained multiplied by a factor between 1 and 0 (1 = full health, 0 = death), which summarizes the quantity and quality of life impact in a single index. The QALYs are used in the a-cost-utility analysis.

#### Willingness to pay

Amount of money that an individual/society is willing to pay to obtain an outcome.
